# Chitin in the Silk Gland Ducts of the Spider *Nephila edulis* and the Silkworm *Bombyx mori*

**DOI:** 10.1371/journal.pone.0073225

**Published:** 2013-08-28

**Authors:** Gwilym J. G. Davies, David P. Knight, Fritz Vollrath

**Affiliations:** Department of Zoology, The University of Oxford, South Parks Road, Oxford, United Kingdom; University of Zurich, Switzerland

## Abstract

Here we report the detection and localisation of chitin in the cuticle of the spinning ducts of both the spider *Nephila edulis* and the silkworm *Bombyx mori*. Our observations demonstrate that the duct walls of both animals contain chitin notwithstanding totally independent evolutionary pathways of the systems. We conclude that chitin may well be an essential component for the construction of spinning ducts; we further conclude that in both species chitin may indicate the evolutionary origin of the spinning ducts.

## Introduction

Silks are a class of proteins spun by arachnids and insects for a variety of biological functions. Of all natural silks the dragline silks of spiders and cocoon silks of silkworms tend to generate most interest; this is because these silks have excellent tensile properties [1], are biocompatible and biodegradable [2]. Importantly, the animals' method of spinning the silk fibre is highly energy efficient in both spiders and silkworms making them valuable models for polymer production [3]. Accordingly, there is considerable interest in how natural silks are formed. A detailed understanding of silk production could assist in the design of artificial methods for spinning high performance protein fibres.

Although considerable effort has been expended in attempts to spin artificial silk, the resulting fibres with few exceptions still fail to match the mechanical properties of their natural inspiration. We argue that this failure is due to an incomplete understanding of the complex processes involved in natural silk production. Therefore, there is a great need for detailed studies of the natural silk ‘production line’ from both a biological and engineering perspective [4].

Silk filaments from silkworms and spiders are produced by largely similar processes [5], [6]. The principal silk proteins have a high molecular weight of around 400 kDa and are secreted by cells lining the long tail of the gland and are stored in the lumen of the silk gland as a concentrated aqueous silk dope solution [7], [8]. When required for spinning, the proteins flow from the gland (through a tapering funnel in the spider [9]) to the spinning duct - a progressively narrowing hyperbolic tube, folded twice in spiders [9] and many times in silkworms [10]. This folding arrangement permits a long duct to fit within the available space [11].

In the spinning duct, the application of strain and shear together with changes in ionic composition along the duct's length are thought to convert the silk dope solution into a solid filament [12]–[14]. At a certain point along the duct, instead of flowing in contact with the duct's inner lining, the silk dope becomes sufficiently gelled to resist tension and pulls away from the wall of the duct in what has been termed an ‘internal drawdown taper’ in both spiders [9] and silkworms [6]. Within this drawdown the protein molecules orient, align and are drawn closer together, expelling water that is subsequently removed by specialised epithelial cells covering the outer surface of the duct's cuticle lining. The removal of water during drawdown helps to initiate the formation of cross-linked beta sheet crystallites interspersed with disordered regions at the molecular level, important for the mechanical properties of silks [5].

Hypotheses regarding the evolutionary origins of the spigot and gland of the spider differ significantly. Previous authors' suggestions for the origin of the spigot and gland fall into one of three categories [15], [16]: first, that the glands evolved *de novo* [15]; second, from dermal layer structures such as from epidermal invaginations [17], limb buds or coxal organs [18], or fluid-secreting setae [19]; and third, from non-dermal structures such as egg sacs [17] as some insect spinning glands appear to be modified salivary glands or malpighian tubules [15]. Importantly, arthropod cuticle, including setae, is largely composed of chitin [20], and here we report on the histochemical detection and localisation of chitin in the cuticle lining of the internal (or internalised) silk gland duct in a spider and silkworm at light microscope level supported by Fourier transform infrared spectroscopy (FTIR) detection of chitin. The study aimed to provide information about the construction of the duct lining relevant to both a consideration of its function and to its evolutionary origin.

## Materials and Methods

### Specimen preparation

*Nephila edulis* specimens were reared at 20°C±5°C. The spiders were fed *Drosophila melanogaster* and *Calliphora vomitaria* flies *ad libitum* and watered twice daily. Experimental specimens weighing from 0.16 g to 1.48 g were anaesthetized by a flow of CO_2_ for five minutes, weighed using an analytical balance (Sartorius BP121S) and imaged at a final magnification of 20× using a digital camera (Canon A640) mounted on a stereo microscope. The width of the cephalothorax at its widest point (the standard metric for spider development [21]) was measured from the digital image with ImageJ software (NIH). Both major ampullate glands and their associated ducts were dissected in spider Ringer solution [22] and gently unfolded to their full length. Spinning *Bombyx mori* silkworms (Padova, Italy and ICIPE, Kenya) were killed with chloroform vapour and the spigot, duct and gland were dissected out in deionised water.

### Histochemical localisation of chitin along the duct

We followed the established protocol for histochemical detection of chitin at the light microscopical level [23], modified to preserve fragile samples [24]. The method involves treatment with KOH to partially deacetylate the chitin and remove non-chitinous matrix components, followed by washing in EtOH, and acidification and staining with iodine. Photomicrographs of the ducts (N = 5 silkworms, 8 spiders) were taken before and after the histochemical reaction, with a digital camera mounted on a stereo microscope. From these photomicrographs, the duct length was measured from tip of spigot to proximal end of funnel with ImageJ software (NIH). From these measurements, the proportion of the length of the duct that remained after the histochemical procedure was calculated.

To search for chitin-containing fragments released during the destruction of the proximal part of the spider's duct the entire histochemical procedure including digestion by KOH, washing in EtOH, acidification and staining was carried out in a silicone grease compressorium (4 ducts from 2 spiders) viewed with bright field illumination (both 10× and 100× oil immersion objective).

The shape of the length of the duct that remained after the histochemical treatment and stained with iodine was estimated by plotting the duct radius profile for the spider size obtained from previous work [25]. This was used to estimate how far along this profile this portion of the duct extended.

Purified powdered chitin from shrimp exoskeleton (Sigma Aldrich) used as positive control stained a deep purple which faded slowly.

### Spectroscopic detection of chitin

For spectroscopic examination, five replicate samples were taken and the mean spectra calculated for each of the following tissues: four silkworms' spigots, head plates and silk gland ducts; the extreme proximal and distal ends of four spiders' ducts (as the proximal third behaved differently to the remainder of the spider duct in the histochemical test), their spigots, cuticles, and midgut. The cells from each were removed by soaking overnight in aqueous 0.1% Tween 40 (Sigma Aldrich) solution on an orbital shaker plate then washed thoroughly in distilled water. To demonstrate the effect of the detergent solution upon the duct, the mean spectra was calculated from four silkworms' ducts rinsed in distilled water rather than Tween solution but otherwise treated identically. We would not expect the effect to be different in spiders as they have remarkably similar ducts [26]. 0.1% Tween 40 was also probed in the same manner.

Spectra were measured on a Nicolet 6700 Fourier transform infrared spectrometer equipped with a liquid nitrogen cooled mercury–cadmium telluride (MCT-A) detector used with a ‘Golden Gate’ single bounce diamond attenuated total reflectance (ATR) sampling accessory (Specac Inc., USA). The diamond's internal reflectance element (IRE) had a refractive index of 2.417 with a 451° angle of incidence. To ensure contact with the sampling window of the spectrometer, the specimen was transferred wet to the ATR cell. Water was then removed under vacuum to leave the sample stuck to the diamond window. Reference samples of commercial purified chitin (Sigma Aldrich) were compressed between the sample window and the anvil of the ATR cell until a good signal level was acquired.

Spectra were analysed with Omnic 7.3 software (Thermo Scientific, USA). The Omnic ‘Correlation’ algorithm which removes offset and eliminates variation in the baseline was used to compare spectra from different specimens.

Any treatment is unlikely to remove all highly bonded protein from chitinous structures, so some protein is likely to remain after the treatment, preventing acquisition of pure chitin spectra but the histochemical method for chitin which uses KOH to remove non-chitinous tissue provides supporting evidence.

## Results and Discussion

### Presence of chitin

#### Spider

**Figure 1:**
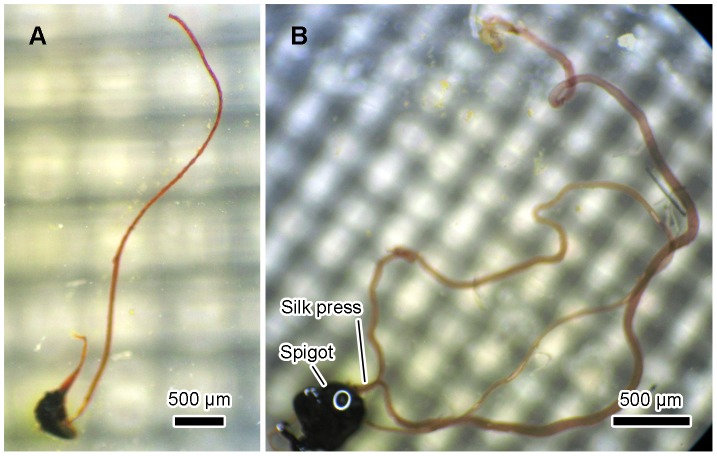
Micrographs of ducts testing positively for chitin. (A) *Nephila edulis*, showing outer cuticle of spinneret and duct, scale bar 500 µm. (B) *Bombyx mori*, showing spigot, silk press then both ducts stained. Scale bar 500 µm.

After performing the histochemical test, whole mounts prepared from the major ampullate duct connected to the spigot showed that the proximal part of the duct had been destroyed leaving only a short length of the distal part of the duct still attached to the spigot, both of which stained a purple/rust red colour (Figure 1 (A)). This colour gradually faded as chitosan is known to be soluble in acetic acid, but the structure itself did not dissolve probably because deacetylation in KOH was only partial [27].

After performing the test extremely gently within the compressorium, we were unable to find any remaining components of the proximal part of the duct within the surrounding fluid even at high magnification.

**Figure 2:**
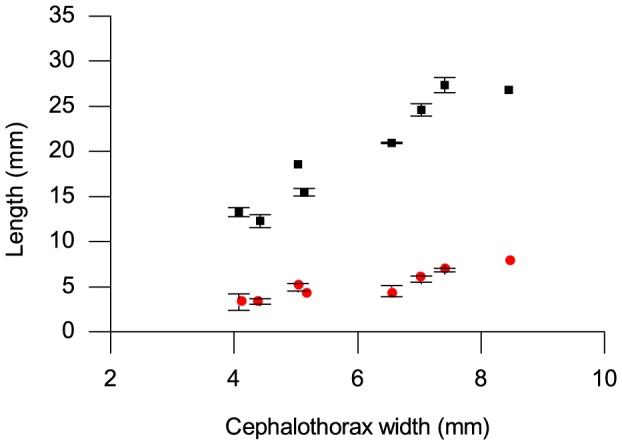
Original length of spider duct and portion testing positive for chitin plotted against cephalothorax width. Original length of duct (black squares), portion testing positive for chitin (red circles). Error bars show standard deviations of length measurements.

The length of the spider duct before the histochemical treatment was a linear function of cephalothorax width (p>0.01; R^2^ = 0.9141, n = 8). After the chitin test, the length of the remaining duct was also a linear function of cephalothorax width (p>0.01; R^2^ = 0.8759, n = 8) (Figure 2).

The proportion of the length of the duct that survived the treatment and stained with iodine remains fairly constant as the spider grows.

#### Silkworm

The entire length of the cuticle lining of the silkworm duct from the distal end of the anterior division of the silk gland to the outer tip of the spigot including the silk press survived KOH treatment and stained purple/rust red colour with iodine in all specimens as illustrated in Figure 1 (B).

#### Location of chitin in spider duct wall relative to duct limbs

**Figure 3:**
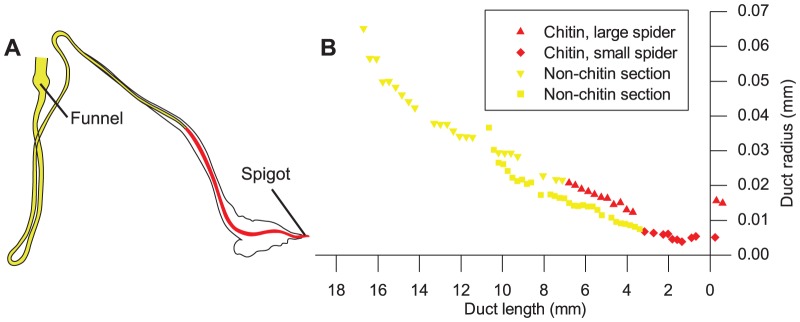
Chitin location within the spider duct. (A) Tracing of spider duct, showing portion solubilised by acid in yellow and section remaining after KOH treatment and testing positive for chitin highlighted in red. (B) Duct profile of both and early and late spider instars from Figure 2 in Davies, Knight et al. 2013, with valve positioned at x = 0. The symbols (red diamonds early instar; red triangles late instar) are for the part of the duct that stains for chitin and the yellow from the remainder of the duct that is destroyed in the test. This indicates that the surviving region is confined to the section of the duct that narrows linearly in both early and late instar spiders.

For each of the spider duct studied, the portion remaining after KOH treatment and staining was found to extend from the tip of the spigot to a point approximately half way along the final limb of the three folded limbs of the duct. This location is shown in Figure 3 (A) in which the portion of duct wall testing positively for chitin is shown superimposed on a traced image of the entire duct.

The graph Figure 3 (B) shows the shape of the ducts in both an early and late spider instar. The section of the duct that tested positively for chitin lies within the part of the duct that decreases linearly in diameter for both early and late instar spiders.

The results from spider ducts indicate that the length of the duct wall staining for chitin increases during organism development and is confined to the linearly narrowing distal section of the duct. Maintenance of the geometry of this part of the duct is thought to be crucial for silk spinning in the spider [25].

### FTIR spectroscopy of ducts

#### Comparisons between the spider proximal and distal duct sections

**Figure 4:**
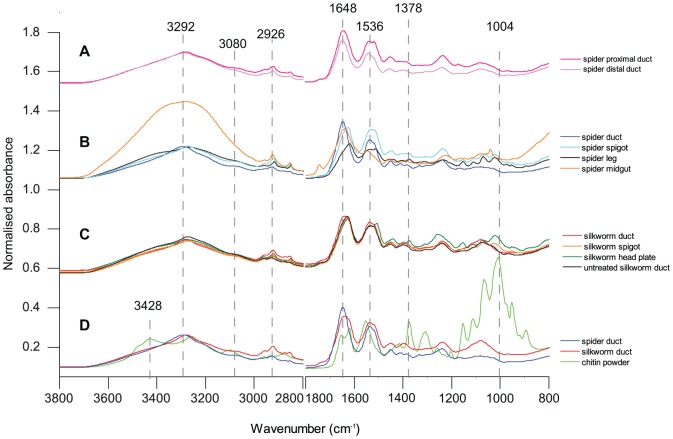
Spectroscopic data with normalised absorbance. (A) Spider proximal and distal ducts, (B) spider duct average, spigot, cuticle and midgut, (C) silkworm duct, spigot, head plate and untreated ducts, (D) spider, silkworm and commercial chitin showing great similarity between results of chitin containing parts of both animals.

The averaged FTIR spectra of the proximal and distal sections of the spider duct shown were similar to each other in shape and scale, with high intensity peaks at 3292 cm^−1^ indicative of the OH bond stretch [28], 3080 and 2926 cm^−1^ indicative of CH symmetric stretching [29], followed by distinctive peaks at 1648 and 1536 cm^−1^ of amide I and amide II [30] (denoted on Figure 4 (A)). A comparison of these peaks to the spectra of chitin is given below. There was little difference between the spectra of the two duct sections, with only a slight decrease in intensity for the distal duct for peaks at wavenumbers below 3080 cm^−1^. The quantitative similarity algorithm in the software calculated a very strong similarity of 94.2% between the two sections of the duct.

#### Comparisons of duct with known chitin-containing spider body parts

Figure 4 (B) shows the averaged spectra acquired from the ducts, cuticle, midgut lining and spigots of the spiders. The only difference (apart from slight differences in intensity of the signal given by the thick and relatively hard cuticle sections compared with the thin lining of soft internal organs) was a slight shift in the amide I peak from 1648 cm^−1^ in the duct to 1619 cm^−1^ in the spider cuticle and to 1626 cm^−1^ in the spigot. The quantitative similarity algorithm calculated an 87.6% similarity between the duct and the spigot, and 58.2% similarity between the duct and the cuticle.

FTIR results for the lining of the midgut in the spider gave a different spectrum to the ducts, cuticle and spigot, with a broad peak at 3239 cm^−1^ due to hydration [28] despite extensive drying of the sample before spectral acquisition. It also showed an additional peak at 1744 cm^−1^ probably due to C = O stretch indicating lipid [31], and a very low intensity amide II band at 1536 cm^−1^. Spectra from the duct and the midgut showed 66.3% similarity. The difference in both hydration and in the carboxyl residue between the spectra of the thoroughly washed and dried midgut and the ducts suggests that the latter structures in the spider are not derived from an internal organ unlike the dermal silk glands present in some insects that are related to the reproductive tract in females [16].

#### Comparison of silkworm duct, spigot and head plate

FTIR of the silkworm duct (Figure 4 (C)) showed a large band at 3279 cm^−1^ indicative of the OH bond, a shoulder at 3080 cm^−1^, a fairly strong band at 2923 cm^−1^ and single amide I and II bands at 1639 and 1537 cm^−1^ respectively, a marked similarity to spectra obtained from silkworm pupal skin [32]. The spectra from the silkworm head plate and spigot were also very similar to both each other and the duct. The spectra from untreated ducts showed the peak at 2926 cm^−1^ to be almost absent, reduced intensity at 1536 cm^−1^ and other smaller peaks, and the peak at 3292 cm^−1^ was more intense. The similarity in mean spectra with treated silkworm ducts was calculated to be only 52.41%, which demonstrates that treatment with detergent had a large effect upon the ducts. The addition spectra from 0.1% Tween 40 and the untreated duct (not shown) showed a similarity of 48.44% with the treated ducts. A comparison of this addition spectra with that of the Tween-extracted duct and the untreated duct suggests that effects of Tween treatment are more likely to result from the removal of cellular materials and the luminal contents of the duct rather than the simple addition of the surfactant.

#### Comparison of silkworm and spider ducts with the chitin reference sample

Figure 4 (D) shows the spectra of the spider and silkworm ducts overlaid with the spectra acquired from powdered commercially available chitin. Although the ducts' single large peak at 3292 cm^−1^ were replaced in the commercial chitin by double peaks at 3428 and 3258 cm^−1^, the shoulder peaks at 3101 and 2877 cm^−1^ were common to both samples. The sharp peak at 1648 cm^−1^ in the ducts was split into a doublet with peaks at 1655 and 1621 cm^−1^ in the commercial chitin and the peak at 1536 cm^−1^ in the latter had shifted to 1553 cm^−1^. In addition, there were a number of peaks including at 1378 and 1004 cm^−1^ in the commercial chitin that were not visible in the duct spectrum.

Averaged spectra from spider duct and silkworm ducts showed 87.8% similarity while commercial chitin powder gave a calculated similarity of 26.3% with spider proximal ducts, 25.3% with spider distal ducts, and 26.8% with silkworm ducts.

The amide I band is used to distinguish between α- and β-chitin, with a doublet of peaks at 1660 and 1627 cm^−1^ in α-chitin, β-chitin a single peak identified at 1656 cm^−1^ [33] or 1650 cm^−1^ [34]. This difference in amide I peaks at 1648 cm^−1^ is attributed to hydrogen bonding between the main chain and side groups in α-chitin, but includes longer bonds that reach between β-sheets in β-chitin [35]. A similar pattern emerges for the part of the spectrum between 3000 and 3500 cm^−1^, in which α-chitin has a shoulder at 3479 cm^−1^ that is not present in β-chitin, and instead has bands at a lower frequency [36]. Comparing the peaks from the ducts to those of resilin, the structural protein associated with chitin in some regions of insect cuticle [37] showed differences in wavenumbers and shape of both the amide I and amide II bands between 1800 and 1200 cm^−1^ [38], [39] but no data outside this region.

The spider and silkworm ducts exhibited many of the hallmarks of β-chitin including high intensity single amide I and II bands at 1648 and 1536 cm^−1^. In highly crystalline forms of β-chitin, the OH band around 3485 cm^−1^ is often weaker or absent but the OH band around 3445 cm^−1^ remains [40], comparable to the single peak at 3292 cm^−1^ in our samples. The spectrum below 1500 cm^−1^ showed very little detail in the spider and silkworm ducts in contrast to the commercial α-chitin, confirming that the use of the amide III bands of this region as an indicator is problematic [41].

We found that the commercial chitin powder had a doublet amide I band with peaks at 1655 and 1621 cm^−1^, a shoulder at 3428 cm^−1^ in addition to a peak at 3258 cm^−1^. Allowing for the variability in the location of the peaks reported in the literature and the frequency shift in spectra obtained from the diamond ATR [42] we identify the commercial sample as containing α-chitin and show that the spider and silkworm ducts contain β-chitin.

### Correlation between chitin histochemistry and FTIR spectroscopy

Both spectroscopic and histochemical tests gave similar results for the spinning ducts of *Nephila edulis* and *Bombyx mori* and the purified reference sample of α-chitin from shrimp shells also gave a positive histochemical reaction for chitin.

As the spectra of the detergent treated spigots and outer cuticle of both animals (composed largely of chitin [20]) were so similar to the spectra of the ducts, and corroborated the histochemical tests for chitin, we conclude that the duct walls of both spider and silkworm contain β-chitin. That the calculated similarity between the spectroscopic results of natural ducts and commercial chitin powder was less than the 85% customarily taken to indicate similarity may be explained by the vigorous extraction methods used in the powdered samples' purification, disrupting their natural molecular structure. The main difference between spectra from commercial chitin and the natural samples was the spectrum below 1500 cm^−1^ already mentioned as problematic, and the increased width of the –OH peak at 3400 cm^−1^, which may depend on the degree of acetylation in chitin from different sources [43] rather than a fundamental difference in composition between the ducts and chitin.

### Chitin presence in silk duct walls

Although the histochemical test appeared to detect chitin only in the distal end of the spider duct, spectroscopy indicated that both the distal end of the duct and the proximal end which dissolved in the KOH both contain chitin and had 94.2% similarity. We suggest that in the proximal part of the spider's duct, the chitin-containing structures are held within a matrix which is broken down by the powerful reagents used in the histochemical test thus releasing the chitinous component(s). We suggest that the distal end of the duct remains intact after digestion either because the matrix is more heavily stabilised or because the chitin nano-components are contiguous rather than encased by a matrix from which they can be released. The difference in matrix between the proximal and distal regions of the duct may relate to subtle differences we observed in their FTIR spectra.

### Relevance of chitin in silk spinning

Pure chitin behaves as a viscoelastic polymer [44], making it very strong, pliable and flexible, whilst being very insoluble in water due to its high crystallinity [45]. However chitin intimately mixed with matrix proteins that are strongly stabilised by hydrophobic interactions and oxidative phenolic tanning can provide very stiff and tough cuticular structures [46]. The drawdown taper in both spiders and silkworms occurs in the distal part of the duct in spiders [9], [47] and silkworms [6], so therefore within the region of chitin that cannot be extracted with KOH in the spider. It is likely that these regions in both spiders and silkworms must be kept stiff to prevent variations in the internal draw down rate (and ratio) brought about by unwanted extensional and/or bending forces applied to the duct lining. These occur for example when the spider spins in a strong wind or when a silkworm bends its head from side to side during spinning [48]. In addition, the start of the draw down taper is just proximal to the clamp in spiders [9], [47] and to the silk press in silkworms [6]. These structures may generate extensional forces in this crucial part of the duct when they clamp down on the silk thread [49]. Thus the arrangement of chitin and heavily stabilised matrix protein in this part of the duct may help to prevent unwanted deformations of this region of the duct whose geometry is thought to be vital for spinning [25].

### Relevance of chitin to origins of silk ducts

Detection of chitin in the cuticle lining of spider and silkworm spinning ducts and the close similarity of the FTIR spectra of these structures with that of exoskeletal structures in the respective species adds credence to the suggestion that silk glands in both glands are dermal in origin. The silk apparatus consisting of a secretory sac connected to a hollow chitin tube, surrounded by ion transporting epithelium and in turn connected to a spigot in essence resembles the ion transporting [50] and fluid secreting trichogen tormogen complex [51] of hollow arthropod spines. The ability of this complex to secrete a non-Newtonian, thigmotactic fluid would have been advantageous to locomotion while selection for an increase in the mechanosensitivity of such a fluid could eventually have led to the ability to form solid silk threads. Thus the spider's silk secreting apparatus may have evolved from simple hollow chitinous spines [15].

Silk worm ducts are thought to be derived from the labial (salivary) glands, with the silk press a combination of the hypopharynx and prementum [52] subsequently modified for silk production [53]. As in the spider this structure may have evolved from the trichogen and tormogen of hollow chitinous spines mounted on the head capsule near the mouth. In this case the non-Newtonian behaviour of the secretion from these spines may have had advantages in sticking together either the food for swallowing or components of a primitive cocoon made from extraneous natural materials and been the starting point for the development of solid threads.

The observation that many of the cuticular proteins associated with chitin in arthropod cuticle are, like silk fibroins, β-sheet proteins [46] supports the trichogen tormogen theory for the origin of arthropod silk glands. Thus silk proteins may have arisen from the ability of certain tormogen cells to secrete silk-like proteins derived from arthropod cuticle into the lumen of the hollow cuticular spines. Further investigations are required to differentiate between the hypotheses presented here and the hypothesis that spiders' silk ducts are lined with chitin due to the developmental constraint of their being attached to the chitinous outer cuticle.

## Conclusion

Histochemical and spectroscopic investigations into the silk ducts of both the spider *Nephila edulis* and the silkworm *Bombyx mori* strongly indicate that they both contain β-chitin, despite an evolutionarily distant common ancestor. Our results suggest that the arrangement of the chitin in the distal end of the duct may be different from that in the proximal end in the spider but not in the silkworm. In both species the chitin-protein composite lining of the duct may help to resist unwanted deformations of the duct wall and highly mechanosensitive silk dope. In both species, the close spectroscopic resemblance of the cuticle lining of the silk duct with exoskeletal structures, and the presence of β-chitin suggests that arthropod silk duct and gland may have evolved from the trichogen and tormogen of a hollow cuticular spine capable of secreting a non-Newtonian fluid derived from a cuticular protein.

## References

[1] Porter D, Guan J, Vollrath F (2012) Spider Silk: Super Material or Thin Fibre? Advanced Materials 25: 1275–1279. 23180482 10.1002/adma.201204158

[2] Altman GH, Diaz F, Jakuba C, Calabro T, Horan RL, et al (2003) Silk-based biomaterials. Biomaterials 24: 401–416. 12423595 10.1016/s0142-9612(02)00353-8

[3] Holland C, Vollrath F, Ryan AJ, Mykhaylyk OO (2012) Silk and Synthetic Polymers: Reconciling 100 Degrees of Separation. Advanced Materials 24: 105–109. 22109705 10.1002/adma.201103664

[4] Vollrath F, Porter D, Holland C (2011) There are many more lessons still to be learned from spider silks. Soft Matter 7: 9595–9600.

[5] Knight DP, Knight MM, Vollrath F (2000) Beta transition and stress-induced phase separation in the spinning of spider dragline silk. International Journal of Biological Macromolecules 27: 205–210. 10828366 10.1016/s0141-8130(00)00124-0

[6] Asakura T, Umemura K, Nakazawa Y, Hirose H, Higham J, et al (2007) Some Observations on the Structure and Function of the Spinning Apparatus in the Silkworm Bombyx mori. Biomacromolecules 8: 175–181. 17206804 10.1021/bm060874z

[7] Prudhomme JC, Couble P (1979) The adaption of the silk gland cell to the production of fibroin in *Bombyx mori* . Biochimie 61: 215–227. 465572 10.1016/s0300-9084(79)80068-1

[8] Sponner A, Schlott B, Vollrath F, Unger E, Grosse F, et al (2005) Characterization of the protein components of Nephila clavipes dragline silk. Biochemistry 44: 4727–4736. 15779899 10.1021/bi047671k

[9] Knight DP, Vollrath F (1999) Liquid crystals and flow elongation in a spider's silk production line. Proceedings of the Royal Society B: Biological Sciences: 519–523.

[10] Akai H (1983) The Structure and Ultrastructure of the Silk Gland. Experientia 39: 443–449.

[11] Bell AL, Peakall DB (1969) Changes in fine structure during silk protein production in ampullate gland of spider Araneus sericatus. Journal of Cell Biology 42: 284–295. 5786985 10.1083/jcb.42.1.284 PMC2107560

[12] Dicko C, Vollrath F, Kenney JM (2004) Spider Silk Protein Refolding Is Controlled by Changing pH. Biomacromolecules 5: 704–710. 15132650 10.1021/bm034307c

[13] Chen X, Knight DP, Shao ZZ, Vollrath F (2002) Conformation transition in silk protein films monitored by time-resolved Fourier transform infrared spectroscopy: Effect of potassium ions on Nephila spidroin films. Biochemistry 41: 14944–14950. 12475243 10.1021/bi026550m

[14] Vollrath F, Knight DP, Hu XW (1998) Silk production in a spider involves acid bath treatment. Proceedings of the Royal Society of London Series B-Biological Sciences 265: 817–820.

[15] Craig CL (1997) Evolution of arthropod silks. Annual Reviews of Entomology 42: 231–267. 10.1146/annurev.ento.42.1.231 15012314

[16] Sutherland T, Young J, Weisman S (2009) Insect Silk: One Name, Many Materials. Annual review of entomology 55: 171–188. 10.1146/annurev-ento-112408-085401 19728833

[17] Kovoor J (1987) Comparative Structure and Histochemistry of silk-producing organs in Arachnids. In: Nentwig W, Ecophysiology of Spiders. Berlin-Heidelberg-New York: Springer. 160–186.

[18] Shultz JW (1987) The origin of the spinning apparatus in spiders. Biological Reviews 62: 89–113.

[19] Young JH, Merritt DJ (2003) The ultrastructure and function of the silk-producing basitarsus in the Hilarini (Diptera: Ernpididae). Arthropod structure & development 32: 157–165. 18089001 10.1016/S1467-8039(03)00006-9

[20] Wainwright SA, Biggs WD, Currey JD, Gosline JM (1976) Mechanical Design in Organisms. New York: John Wiley and Sons.

[21] Hagstrum DW (1971) Carapace width as a tool for evaluating rate of development of spiders in laboratory and field. Annals of the Entomological Society of America 64: 757.

[22] Groome JR, Townley MA, Detschaschell M, Tillinghast EK (1991) Detection and isolation of proctolin-like immunoreactivity in arachnids - possible cardioregulatory role for proctolin in the orb-weaving spiders argiope and araneus. Journal of Insect Physiology 37: 9–19.

[23] Campbell FL (1929) The detection and estimation of insect chitin; and the irrelation of “chitinization” to hardness and pigmentation of the cuticula of the American cockroach, Periplaneta americana L. Annals of the Entomological Society of America 22: 401–426.

[24] Ravindranath MHR, Ravindranath MH (1975) A simple procedure to detect chitin in delicate structures. Acta Histochemica 53: 203–205. 811052

[25] Davies GJG, Knight DP, Vollrath F (2013) Structure and Function of the Major Ampullate Spinning Duct of the Golden Orb Weaver, *Nephila edulis*. Tissue and Cell. 10.1016/j.tice.2013.04.001 23664309

[26] Vollrath F, Knight DP (2001) Liquid crystalline spinning of spider silk. Nature 410: 541–548. 11279484 10.1038/35069000

[27] Peters W (1992) Peritrophic Membranes: Springer.

[28] Libowitzky E, Rossman GR (1997) An IR absorption calibration for water in minerals. American Mineralogist 82: 1111–1115.

[29] Ibrahim M, Nada A, Kamal DE (2005) Density functional theory and FTIR spectroscopic study of carboxyl group. Indian Journal of Pure & Applied Physics 43: 911–917.

[30] Barth A, Zscherp C (2002) What vibrations tell us about proteins. Quarterly Reviews of Biophysics 35: 369–430. 12621861 10.1017/s0033583502003815

[31] Nzai JM, Proctor A (1999) Soy lecithin phospholipid determination by Fourier transform infrared spectroscopy and the acid digest arseno-molybdate method: A comparative study. Journal of the American Oil Chemists Society 76: 61–66.

[32] Paulino AT, Simionato JI, Garcia JC, Nozaki J (2006) Characterization of chitosan and chitin produced from silkworm crysalides. Carbohydrate Polymers 64: 98–103.

[33] Cardenas G, Cabrera G, Taboada E, Miranda SP (2004) Chitin characterization by SEM, FTIR, XRD, and C-13 cross polarization/mass angle spinning NMR. Journal of Applied Polymer Science 93: 1876–1885.

[34] Jang MK, Kong BG, Jeong YI, Lee CH, Nah JW (2004) Physicochemical characterization of alpha-chitin, beta-chitin, and gamma-chitin separated from natural resources. Journal of Polymer Science Part A-Polymer Chemistry 42: 3423–3432.

[35] Tanner SF, Chanzy H, Vincendon M, Roux JC, Gaill F (1990) High-resolution solid-state C-13 nuclear-magnetic-resonance study of chitin. Macromolecules 23: 3576–3583.

[36] Focher B, Naggi A, Torri G, Cosani A, Terbojevich M (1992) Chitosans from Euphausia-superba 2. Characterization of solid-state structure. Carbohydrate Polymers 18: 43–49.

[37] Weis-Fogh T (1960) A Rubber-Like Protein in Insect Cuticle. Journal of Experimental Biology 37: 889–907.

[38] Tamburro AM, Panariello S, Santopietro V, Bracalello A, Bochicchio B, et al (2010) Molecular and Supramolecular Structural Studies on Significant Repetitive Sequences of Resilin. ChemBioChem 11: 83–93. 19943267 10.1002/cbic.200900460

[39] Qin G, Hu X, Cebe P, Kaplan DL (2012) Mechanism of resilin elasticity. Nature Communications 3.. 10.1038/ncomms2004 PMC3527747 22893127

[40] Parker KD, Rudall KM (1957) Structure of the Silk of Chrysopa Egg-stalks. Nature 179: 905–906.

[41] Jackson M, Mantsch HH (1995) The Use and Misuse of FTIR Spectroscopy in the Determination of Protein-Structure. Critical Reviews in Biochemistry and Molecular Biology 30: 95–120. 7656562 10.3109/10409239509085140

[42] Boulet-Audet M, Buffeteau T, Boudreault S, Daugey N, Pezolet M (2010) Quantitative Determination of Band Distortions in Diamond Attenuated Total Reflectance Infrared Spectra. Journal of Physical Chemistry B 114: 8255–8261. 10.1021/jp101763y 20507143

[43] Muzzarelli RAA (1977) Chitin. Oxford: Pergamon Press. 309.

[44] Ruiz-Herrera J, Martinez-Espinoza AD (1999) Chitin biosynthesis and structural organization *in vivo*. In: Jollès P, Muzzarelli RAA, Chitin and Chitinases: Springer. 10.1007/978-3-0348-8757-1_3 10906950

[45] Pillai CKS, Paul W, Sharma CP (2009) Chitin and chitosan polymers: Chemistry, solubility and fiber formation. Progress in Polymer Science 34: 641–678.

[46] Vincent JFV (2012) Structural Biomaterials. 3rd ed. Princeton, New Jersey, and Woodstock, UK: Princeton University Press. 129–132.

[47] Vollrath F, Knight DP (1999) Structure and function of the silk production pathway in the spider Nephila edulis. International Journal of Biological Macromolecules 24: 243–249. 10342771 10.1016/s0141-8130(98)00095-6

[48] Magoshi J, Magoshi Y, Nakamura S (1994) Mechanism of Fiber Formation of Silkworm. In: Kaplan DL, Adams WW, Viney C, Farmer BL, Silk Polymers: Materials Science and Biotechnology. Washington, DC: American Chemical Society. 292–310.

[49] Fu CJ, Shao ZZ, Fritz V (2009) Animal silks: their structures, properties and artificial production. Chemical Communications: 6515–6529. 10.1039/b911049f 19865641

[50] Habel RE (1963) Carbohydrates, phosphatases, and esterases in mucosa of ruminant forestomach during postnatal development. American Journal of Veterinary Research 24: 199. 13951627

[51] Phillips CE, Vandeberg JS (1976) Mechanism for sensillum fluid-flow in trichogen and tormogen cells of *Phormia-regina* (meigen) (Diptera-Calliphoridae). International Journal of Insect Morphology & Embryology 5: 423–431.

[52] Snodgrass RE (1935) Principles of insect morphology. ix+667p. 319 fig.-ix+667p. 319 fig. p.

[53] Yonemura N, Sehnal F (2006) The Design of Silk Fiber Composition in Moths Has Been Conserved for More Than 150 Million Years. Journal of Molecular Evolution 63: 42–43. 16755355 10.1007/s00239-005-0119-y

